# Effects of low-volume walking programme and vitamin E supplementation on oxidative damage and health-related variables in healthy older adults

**DOI:** 10.1186/1743-7075-10-38

**Published:** 2013-05-09

**Authors:** Jong-Hwan Park, Masashi Miyashita, Masaki Takahashi, Noriaki Kawanishi, Seong-Ryu Bae, Hyun-Shik Kim, Katsuhiko Suzuki, Yoshio Nakamura

**Affiliations:** 1 Graduate School of Sport Sciences, Waseda University, 2-579-15 Mikajima, Tokorozawa, Saitama 359-1192, Japan; 2 Department of Health and Sports Sciences, Tokyo Gakugei University, 4-1-1 Nukuikitamachi, Koganei 184-8501, Japan; 3 Research Fellow of the Japan Society for the Promotion of Science, 6 Ichibancho, Chiyoda-ku, Tokyo 102-8471, Japan; 4 Faculty of Sport Sciences, Waseda University, 2-579-15 Mikajima, Tokorozawa, Saitama 359-1192, Japan

**Keywords:** Low-volume physical activity, Vitamin E, Oxidative damage, Older adults

## Abstract

**Background:**

Both exercise and vitamin E supplementation have been shown to reduce oxidative stress and cardiovascular disease risk in older adults, and when combined there is evidence suggesting that they act synergistically. The currently recommended amount of exercise for older adults is 150 min/week of moderate-intensity exercise; however, the minimum amount of exercise necessary to achieve health benefits is not known. The purpose of this study was to investigate the effects of 12 weeks of participation in a low-volume walking exercise programme (i.e. 90 min/week) combined with daily vitamin E supplementation on thiobarbituric acid reactive substances (TBARS) and oxidised low-density lipoprotein (LDL) concentrations in older adults.

**Methods:**

The participants were recruited from the following four groups separately: 1) control (CG, n = 14), 2) vitamin E supplementation (SG, n = 10), 3) walking (WG, n = 7), or 4) walking + supplementation (WSG, n = 7). In the CG, participants were advised to maintain their normal lifestyle during the study. Participants in both the SG and WSG received 450 IU (300 mg) /day of α-tocopherol for 12 weeks. The exercise programme for the WG and WSG consisted of two 30–60 minute sessions weekly for 12 weeks (average walking time was 44.5 ± 1.6 min/session). Blood samples were collected at baseline and at 12 weeks.

**Results:**

Delta plasma oxidised LDL concentrations did not differ among four groups (One-factor ANOVA, *P* = 0.116). However, negative delta plasma TBARS, a marker of oxidative damage, concentrations were observed in the WG, WSG and SG relative to the CG at the end of the study period (One-factor ANOVA, *P* = 0.001; post hoc tests; CG compared with WG, WSG and SG, *P* = 0.005; *P* = 0.021; *P* = 0.024, respectively).

**Conclusion:**

These findings suggest that a low-volume of physical activity and/or vitamin E supplementation may be an effective intervention strategy for reducing TBARS concentrations of older adults.

**Trial registration:**

UMIN000008304

## Background

Oxidative stress leads to vascular dysfunction and has been implicated in the pathogenesis of aging and atherosclerosis [1]. The oxidative modification of low-density lipoprotein (LDL) is a key step in the initiation and progression of atherosclerosis [2]. Uptake of oxidised LDL by macrophages via scavenger receptors leads to lipid-laden foam cell formation and fatty streak development in the arterial wall [3]. In addition, oxidised LDL, oxidative stress, and markers of chronic inflammation increase with advancing age [4,5]. Identifying effective strategies for preventing the oxidative modification of LDL and oxidative stress in older adults may reduce cardiovascular disease risk, and thus is an important public health issue.

It has been previously reported that plasma concentrations of oxidised LDL and triacylglycerol were reduced after high intensity training (66% of heart rate reserve, 60 minutes/session, 3 times/week for 10 weeks) in older adults [6]. However, it is important to note that it is difficult to achieve compliance when recommending high-intensity physical activity to older adults. In addition, the optimal amount of exercise for achieving health benefits is still unknown, and may be lower than the currently recommended level of 150 minutes/week of moderate-intensity exercise [7]. To develop effective physical activity strategies to reduce cardiovascular disease risks in older adults, it is important to further identify the effects of lower amounts of moderate-intensity physical activity (such as walking) on markers of cardiovascular disease risk, including insulin, glucose and high-density lipoprotein (HDL)-cholesterol.

There is an inverse relationship between vitamin E intake and the prevalence of coronary heart disease [8,9]. In particular, α-tocopherol (an active form of vitamin E), exhibits antioxidant and anti-inflammatory effects and reduces the risk of atherosclerosis and cardiovascular disease [10,11]. However, there is inconsistency in the literature. A previous study has demonstrated that 3 years of vitamin E supplementation (i.e. 136 IU of dl-α-tocopherol (91 mg)) did not influence oxidative stress in hypercholesterolaemic women patients [12]. Whereas another previous study has indicated that 16 weeks of moderate-intensity endurance exercise training with high dose of vitamin E (i.e. 800 IU of dl-α-tocopherol (534 mg)) daily intake reduces lipid hydroperoxide (a marker of oxidative stress) and resting blood pressure in older adults [13]. Contrastively, a study of Bobeuf et al. investigated that the combination of 6 month of resistance training and antioxidant supplementation (i.e. 1000 mg of vitamin C and 400 IU of vitamin E (267 mg)) had a positive effect on the plasma antioxidant profile but not on the oxidative stress in older adults [14]. These previous studies remain unclear whether exercise programme and/or vitamin E supplementation in the older adults has a positive effect on the oxidative stress and oxidised LDL concentrations.

Therefore, the main purpose of this study was to examine the effects of 12 weeks of participation in a low-volume walking programme combined with vitamin E supplementation on concentrations of thiobarbituric acid reactive substances (TBARS, a marker of oxidative damage) and oxidised LDL in older adults.

## Methods

### Participants

Participants aged 60–81 years were recruited from the local communities. For the baseline evaluation, we used a simple lifestyle-related questionnaire (physical activity, medication, sleep, alcohol intake, and smoking). Patients with history of cardiovascular disease, physically active lifestyle, those who used lipid- and/or glucose-lowering medication, were smokers, or <60 years of age were excluded from the study. A physically active lifestyle was defined as any form of planned, structured, and repetitive physical activity undertaken a minimum of 3 times per week, lasting at least 30 min per session, for the purpose of improving or maintaining one or more components of physical fitness described by Butcher et al. [15]. Consequently, none of the study participants were trained athletes in any sporting events but some participants were recreationally active. We recruited forty-two participants (10 males and 32 females) who initially met the criteria for the enrollment in the present study. From an ethical point of view, we have considered that we should not randomly assign the participants to each group, particularly those who do not wish to increase/decrease their activities of daily living or receive dietary supplements - we have recruited the participants of each group at the same time and thus each participant knows what they are volunteering for. Fourteen older adults were recruited for the control group (CG). Ten older adults were recruited for the vitamin E supplementation group (SG). Seven older adults were recruited for the walking group (WG). Seven older adults were recruited for the walking and vitamin E supplementation group (WSG). Four participants (1 male and 3 females) in the WG could not complete the walking program of the present study because of injury or disease (i.e. not related to the present study). Consequently, thirty-eight participants (9 males and 29 females) were included in the analysis. Each participant was instructed not to change their lifestyle, including dietary habits, throughout the study period. Participants of the control group were advised to maintain their normal lifestyle during the study. The physical characteristics of the participants are shown in Table 1. This study was approved by the Waseda University Ethical Advisory Committee (2011–061). Informed consent was obtained from all participants.

**Table 1:** Changes in body composition at baseline and after 12 weeks

		**Groups**				***P *- value**
		**WG (*N *= 7)**	**WSG (*N *= 7)**	**SG (*N *= 10)**	**CG (*N *= 14)**	**(time, interaction)**
Sex, male/female	Baseline	1/6	0/7	2/8	6/8	-
Age (years)	Baseline	71.9 ± 1.9	65.9 ± 2.0	67.0 ± 1.8	71.9 ± 1.6	-
Height (m)	Baseline	1.54 ± 0.02	1.55 ± 0.02	1.57 ± 0.02	1.57 ± 0.02	-
Body mass (kg)	Baseline	53.98 ± 3.17	58.51 ± 3.27	52.56 ± 2.21	54.71 ± 2.14	0.020, 0.263
	12 weeks	54.69 ± 3.28†	58.30 ± 3.40	53.07 ± 2.28†	55.41 ± 2.06	
	Δ	+0.71 ± 0.28	−0.21 ± 0.51	+0.51 ± 0.15	+0.70 ± 0.33	
BMI (kg/m^2^)	Baseline	22.67 ± 1.02	24.42 ± 1.48	21.37 ± 0.65	22.17 ± 0.53	0.024, 0.286
	12 weeks	22.95 ± 1.03	24.33 ± 1.55	21.57 ± 0.66†	22.45 ± 0.45	
	Δ	+0.29 ± 0.12	−0.85 ± 0.21	+0.20 ± 0.56	+0.28 ± 0.14	
Waist circumference (cm)	Baseline	81.07 ± 3.10	84.44 ± 2.84	80.76 ± 2.50	78.84 ± 1.76	0.603, 0.194
	12 weeks	84.20 ± 2.71†	82.19 ± 3.79	80.13 ± 2.10	80.39 ± 2.12	
	Δ	+3.13 ± 1.16	−2.24 ± 2.45	−0.63 ± 2.04	+1.55 ± 1.04	
SBP (mm Hg)	Baseline	136.4 ± 4.2	139.7 ± 4.2	120.2 ± 3.1	135.7 ± 3.4	0.265, 0.547
	12 weeks	138.4 ± 4.8	137.4 ± 3.6	124.2 ± 3.1	139.5 ± 2.0	
	Δ	+2.0 ± 4.0	−2.3 ± 4.3	+4.0 ± 3.0	+3.8 ± 2.4	
DBP (mm Hg)	Baseline	78.9 ± 3.0	83.7 ± 3.8	73.2 ± 1.9	79.4 ± 2.5	0.737, 0.639
	12 weeks	79.0 ± 3.8	81.0 ± 3.3	72.3 ± 1.4	81.1 ± 2.0	
	Δ	+0.1 ± 3.0	−2.7 ± 3.4	−0.9 ± 1.5	+1.7 ± 2.2	

Values are mean ± SEM. WG, walking group; WSG, walking + supplementation group; SG, supplementation group; CG, control group; BMI, body mass index; SBP, systolic blood pressure; DBP, diastolic blood pressure.

† Significantly different from the baseline value in the same group (Paired *t*-test, *P* < 0.05).

Δ, 12 weeks – baseline difference; w, walking; ws, walking + supplementation; s, supplementation; c, control.

### Walking programme

All participants in the WG and WSG underwent a 12-week, supervised exercise programme that consisted of 2 walking exercise sessions per week. Each 30–60 minute walking session was done in the morning, between 10:00 and 11:00, and included 5 minutes of warm-up activities and 5 minutes of cool-down activities. During weeks 1–5, participants walked 2.6–3.5 km in 30–40 minute walking sessions. During weeks 6–12, participants completed 40–60 minute walking sessions covering distances of 3.5–4.9 km. All walking sessions were performed outdoors and were supervised by experienced assistants. The participants were asked to indicate their perceived exertion during each walking session using the Borg scale [16], and heart rate was measured using short-range telemetry (Polar RS400, Polar Electro Oy, Finaland). Participants in all groups were advised to maintain their usual daily living activities during the study.

### Vitamin E supplementation

Participants in the SG and WSG were issued a 12-week supply of 150 IU (100 mg) vitamin E (RRR α-tocopherol) (Juvelux 300, Eisai Co., Ltd., Japan) capsules and instructed to take one-capsule thrice daily (RRR α-tocopherol, 450 IU (300 mg) /day) with meals for the entire 12 week study period.

### Physical activity measurement

To determine the general physical activity level of the participants, all of the participants were asked to wear a uniaxial accelerometer (Lifecoder-EX, Suzuken Co. Ltd., Nagoya, Japan) for the 12 week study period. This device determines the level of activity intensity by measuring the magnitude and frequency of accelerations every 4 seconds. Activity intensity is classified into one of eleven levels (0, 0.5, 1–9), with 0 indicating the lowest and 9 indicating the highest level of activity. Participants were contacted by phone at the beginning of each week to assess compliance and any device problems, and all reported that there were no problems with the use of the accelerometer. Our data analysis confirmed that all participants wore the accelerometer every day during the data collection period. Since we collected accelerometer data from each group simultaneously, seasonality was not an issue in the present study. Data from participants who had worn the accelerometer for at least 10 hours a day for at least 4 weekdays and 1 weekend day after calculation of wear time were considered valid [17,18]. The main physical activity variable used in this study was the time spent in moderate to vigorous physical activity (MVPA). The number of minutes spent performing MVPA was calculated on a daily basis, and then used to estimate weekly activity by taking a weighted average of daily weekday and weekend activity (i.e., weekly MVPA = (average daily weekday MVPA × 5) + (average daily weekend MVPA × 2)). All minutes of recording with a total of ≥ 4 activity levels were classified as MVPA. The activity level score of 4 was utilised as a threshold for MVPA based on a previous calibration study [19] and corresponded to approximately 3 metabolic equivalents.

### Anthropometric and blood pressure measurements

Anthropometric variables were measured at baseline and after 12 weeks in each group. Body mass was measured to the nearest 0.05 kg using a digital scale (Inner Scan 50, Tanita Corporation, Tokyo, Japan). Height was measured to the nearest 0.1 cm using a wall-mounted stadiometer (YS-OA, As One Corporation, Osaka, Japan). Body mass index (BMI) was calculated as weight in kilograms divided by the square of height in meters. Waist circumference was measured to the nearest 0.1 cm at the level of the umbilicus using a flexible plastic tape while the participants were in a standing position. Arterial blood pressure was measured using a mercury sphygmomanometer (605P, Yagami Co. Ltd., Nagoya, Japan) after participants had been seated at rest for 5 minutes. For each variable, two measurements were taken at each time point and the mean of these values was recorded.

### Dietary assessment

To examine whether dietary intake of antioxidants influenced outcome factors, diet recall questions were conducted at baseline and during week 10 of the study period. Diet records were analysed using a computerised nutritional analysis system (Excel Eiyokun, Kenpakusha, Japan). Dietary assessments were conducted by a registered dietitian.

### Blood collection and laboratory assays

Fasting venous blood samples were taken from an antecubital vein at baseline and at 12 weeks. Participants were asked to avoid physical activity for 48 hours, and to fast overnight for at least 10 hours, before the blood samples were taken. Blood concentrations of oxidised LDL, thiobarbituric acid reactive substances (TBARS), α-tocopherol, glucose, insulin, HbA1c, soluble E-selectin (sE-selectin), soluble vascular cell adhesion molecule (sVCAM-1), C-peptide, triacylglycerol, total cholesterol (TC), HDL-cholesterol and low-density lipoprotein (LDL)-cholesterol were measured. For α-tocopherol, triacylglycerol, TC, HDL-cholesterol and LDL-cholesterol, the blood samples were collected into tubes containing clotting activators to facilitate separation of serum. The samples were allowed to clot for 45 minutes at room temperature and were then centrifuged at 3000 rpm for 10 minutes at 4°C. After separation, serum was aliquoted into plain micro tubes and stored at −80°C for later analysis. For HbA1c, oxidised LDL, TBARS, insulin, sE-selectin, sVCAM-1 and C-peptide, venous blood samples were collected into tubes containing disodium salt-EDTA. The samples were immediately centrifuged and the plasma separated and aliquoted as described above. For glucose measurements, venous blood samples were collected into tubes containing sodium fluoride-EDTA, immediately centrifuged, and aliquoted as described above.

Plasma concentrations of oxidised LDL (Mercodia AB, Uppsala, Sweden), insulin (Mercodia AB, Uppsala, Sweden), sE-selectin (eBioscience, Inc., San Diego, USA), sVCAM-1 (eBioscience, Inc., San Diego, USA) and C-peptide (Mercodia AB, Uppsala, Sweden) were measured by enzyme-linked immunosorbent assay using commercially available kits. Concentrations of serum triacylglycerol, TC, HDL-cholesterol, LDL-cholesterol, plasma glucose and HbA1c were determined using standard laboratory methods. Plasma TBARS was measured using an assay kit from Cayman Chemicals (Cayman Chemicals, Michigan, USA). The concentration of serum α-tocopherol was measured using high performance liquid chromatography (HPLC) by the method of Talwar et al. [20]. Then, serum α-tocopherol concentrations were normalised to serum triacylglycerol and TC concentrations.

### Statistical analysis

Data were analysed using Predictive Analytics Software (PASW) version 18.0 for Windows (SPSS Japan Inc., Tokyo, Japan). The Shapiro-Wilk test was used to check for normality of distribution. One-factor analysis of variance (ANOVA) was used to examine the differences in the baseline and delta values of physical and physiological variables, dietary and physical activity data, and blood markers among the 4 groups. A two-factor ANOVA was used to determine differences among groups over time for all outcome variables. Where significant effects were found, post hoc pair-wise comparisons were performed using the Bonferroni method. Paired Student’s t-tests were used to assess group differences between pre- and post-intervention data. Statistical significance was accepted at the 5% level. Results are presented as means ± SEM.

## Results

### Physical characteristics and dietary data

**Figure 1:**
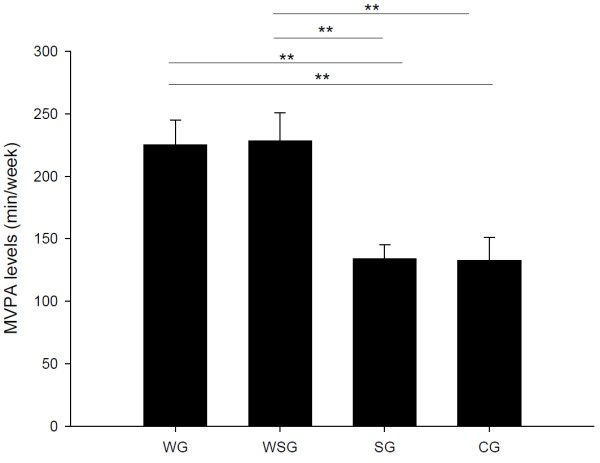
**Mean (± SEM) moderate to vigorous physical activity (MVPA) levels in the control group (CG), vitamin E supplementation group (SG), walking group (WG) and vitamin E plus walking group (WSG).** **Significantly different from the between group, P ≤ 0.015.

There were no between-group differences in physical and physiological characteristics at baseline, with the exception of systolic blood pressure (SBP) (Table 1). One-factor ANOVA revealed a significant baseline difference in SBP among groups (*P* = 0.004). SBP was significantly lower in the SG (120.3 ± 3.1 mm Hg) than in the CG (135.7 ± 3.4 mm Hg, *P* = 0.016), WG (136.4 ±4.2 mm Hg, *P* = 0.045), and WSG (139.7 ± 4.2 mm Hg, *P* = 0.010). Within-group analyses showed that body mass increased significantly in the SG (paired *t*-test, *P* = 0.008) and WG (paired *t*-test, *P* = 0.045) after 12 weeks relative to their baseline values, while BMI increased only in the SG (paired *t*-test, *P* = 0.006) and waist circumference increased only in the WG (paired *t*-test, *P* = 0.035) (Table 1). Figure 1 shows the MVPA levels measured during the 12 weeks period among the four groups. One-factor ANOVA revealed a significant difference in MVPA measured during the 12 weeks period among groups (*P* = 0.001). Post hoc tests showed that MVPA measured during the 12 week study period was significantly higher in the WG (225.1±19.7 min/week) and WSG (228.2± 22.8 min/week) than in the CG (132.7±18.2 min/week) and SG (134.0± 11.3 min/week). Analysis of dietary data revealed that dietary intake (excluding supplementation) of the antioxidants vitamin C, vitamin E and β-carotene did not differ between or within groups at baseline or during the study.

### Exercise compliance and exercise amount

The mean duration of the walking sessions was 44.5 ± 1.6 min/session. The mean heart rate and perceived exertion during the walking sessions was 113 ± 3 beats/min and 10.6 ± 0.3 (i.e. light), respectively. This corresponded to an exercise intensity of 48 ± 4% of heart rate reserve.

### Serum α-tocopherol concentrations

**Figure 2:**
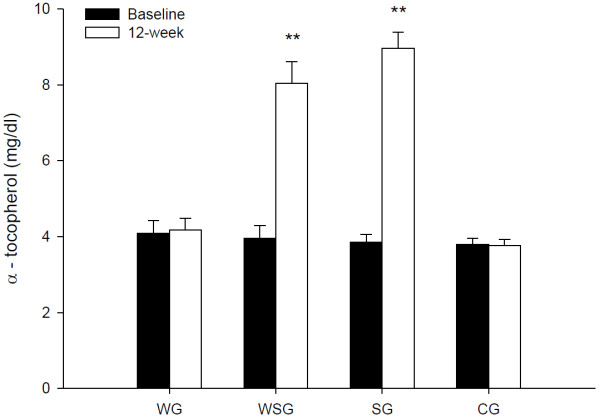
**The serum α-tocopherol concentrations (normalised for triacylglycerol and total cholesterol concentrations) measured at baseline and at 12 weeks in the control group (CG), vitamin E supplementation group (SG), walking group (WG) and vitamin E plus walking group (WSG).** Data are means ± SEM and time-group interactions are the main influencing factors (*P* = 0.001, Two-factor ANOVA). **Significantly different from the baseline value in the same group (paired Student’s *t* test, P < 0.01).

Figure 2 shows the serum α-tocopherol concentrations measured at baseline and at 12 weeks in the CG, SG, WG and WSG. At baseline, there were no differences in serum α-tocopherol concentrations among groups. Two-factor ANOVA revealed that there were main effects of time (*P* = 0.001) and time by group interactions (*P* = 0.001) for serum α-tocopherol concentrations.

### Plasma oxidised LDL and TBARS responses

**Figure 3:**
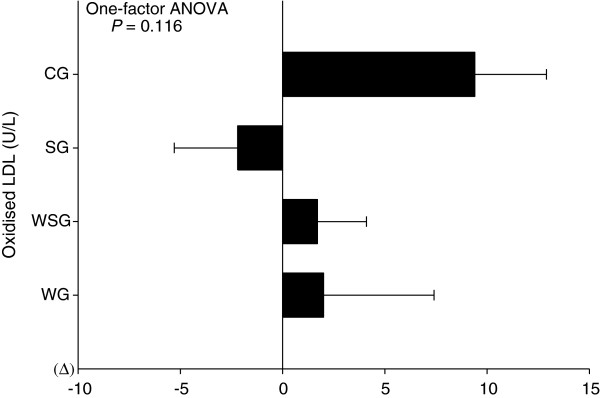
**Delta value of plasma thiobarbituric acid reactive substances (TBARS) measured at baseline and at 12 weeks in the control group (CG), vitamin E supplementation group (SG), walking group (WG) and vitamin E plus walking group (WSG).** Data are means ± SEM. Data were analysed using one-factor ANOVA followed by a Bonferroni multiple comparisons test (*P* = 0.001, One-factor ANOVA). ** Significantly different from the control group (*P* ≤ 0.024).

**Figure 4:**
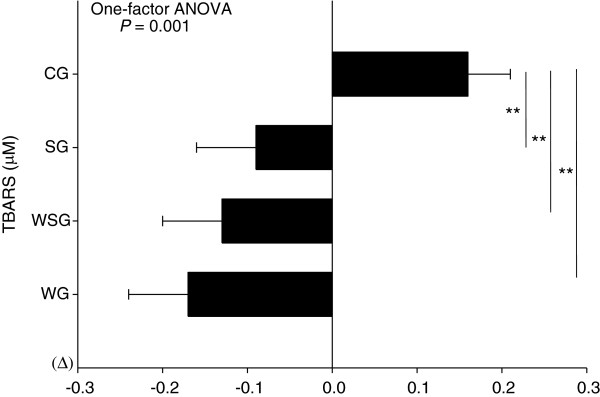
**Delta value of oxidised low-density lipoprotein (LDL) concentrations measured at baseline and 12 weeks in the control group (CG), vitamin E supplementation group (SG), walking group (WG) and vitamin E plus walking group (WSG).** Data are means ± SEM. Data were analysed using one-factor ANOVA followed by a Bonferroni multiple comparisons test (*P* = 0.116, One-factor ANOVA). There were no significant differences among group.

Two-factor ANOVA revealed that there were no main effects of time (*P* = 0.172) and no time by group interactions (*P* = 0.116) for plasma oxidised LDL concentrations. One-factor ANOVA revealed that changes in plasma oxidised LDL concentrations did not differ among groups (*P* = 0.116) (Figure 3). However, within-group analysis showed that plasma oxidised LDL concentrations significantly increased in the CG (*P*= 0.019) over the 12 week study period. For plasma TBARS concentrations, two-factor ANOVA revealed that there was no main effect of time (*P* = 0.083), but there was a significant time by group interaction (*P* = 0.007). Plasma TBARS concentration was significantly greater at 12 weeks in the CG compared with the WG, WSG and SG (post hoc tests; CG compared with WG, WSG and SG, P = 0.005; P = 0.021; P = 0.024, respectively) (Figure 4). Within-group analyses showed that plasma TBARS concentrations significantly decreased over the study period only in the WG (*P* = 0.038), and increased only in the CG (*P* = 0.042), relative to baseline values.

### Biochemical and hormonal parameters

Biochemical and hormonal parameters at baseline and after 12 weeks are presented in Table 2. For serum TC concentrations, two-factor ANOVA revealed that there were main effects of time (*P* = 0.001) and time by group interactions (*P* = 0.012). Within-group analyses showed that serum TC concentrations were significantly increased in the WG, WSG and SG (paired *t*-test, *P* = 0.004; *P* = 0.048; *P* = 0.043, respectively) after 12 weeks relative to baseline values. Two-factor ANOVA revealed that there were no significant time by group interactions (*P* ≥ 0.060) for serum HDL-cholesterol, plasma HbA1c, sE-selectin, sVCAM-1 and C-peptide concentrations. Within-group analyses showed that serum HDL-cholesterol concentrations significantly increased in the WG (paired *t*-test, *P* = 0.038), plasma HbA1c concentrations significantly decreased in both the WSG and SG (paired *t*-test, *P* = 0.001; *P* = 0.023, respectively), and plasma glucose, sE-selectin, sVCAM-1, and C-peptide concentrations significantly increased in the CG (paired *t*-test, *P* = 0.045; *P* = 0.004; *P* = 0.002; *P* = 0.001, respectively) over the 12 week study period (Table 2). No significant changes were observed in serum triacylglycerol, serum LDL-cholesterol and plasma insulin concentrations between or within the groups.

**Table 2:** Changes in blood parameters at baseline and after 12 weeks

		**Groups**				***P *- value**
		**WG (*N *= 7)**	**WSG (*N *= 7)**	**SG (*N *= 10)**	**CG (*N *= 14)**	**(time, interaction)**
Triacylglycerol (mg/dl)	Baseline	100.9 ± 13.1	97.3 ± 11.6	85.0 ± 6.0	99.1 ± 15.0	0.109, 0.703
	12 weeks	112.7 ± 15.8	106.0 ± 13.1	99.0 ± 13.6	99.1 ± 12.4	
	Δ	+11.9 ± 15.9	+8.7 ± 8.9	+14.0 ± 1.0	−0.1 ± 7.4	
TC (mg/dl)	Baseline	199.1 ± 9.8	224.7 ± 13.6	201.7 ± 6.8	212.6 ± 5.7	0.001, 0.012
	12 weeks	235.3 ± 10.7†	237.6 ± 11.4†	217.5 ± 7.3†	214.6 ± 7.1	
	Δ	+36.1 ± 8.1‡	+12.9 ± 5.2	+15.8 ± 6.7	+2.1 ± 6.1	
HDL-C (mg/dl)	Baseline	61.6 ± 4.0	64.7 ± 7.6	56.3 ± 3.4	63.5 ± 5.35	0.081, 0.165
	12 weeks	70.6 ± 6.6†	63.7 ± 7.0	59.1 ± 3.7	63.8 ± 4.15	
	Δ	+9.0 ± 3.4	−1.0 ± 3.0	+2.8 ± 1.3	+0.3 ± 3.2	
LDL-C (mg/dl)	Baseline	115.0 ± 7.1	132.6 ± 11.2	119.4 ± 6.3	118.7 ± 5.4	0.004, 0.122
	12 weeks	135.0 ± 9.1	140.3 ± 9.1	125.6 ± 7.6	119.4 ± 5.8	
	Δ	+20.0 ± 8.4	+7.7 ± 5.5	+6.2 ± 5.5	+0.7 ± 3.8	
Glucose (mg/dl)	Baseline	96.9 ± 2.8	110.0 ± 8.4	91.1 ± 1.5	90.2 ± 1.2	0.992, 0.098
	12 weeks	100.3 ± 2.9	106.1 ± 8.8	89.4 ± 2.2	92.8 ± 1.1†	
	Δ	+3.4 ± 2.7	−3.9 ± 3.1	−1.7 ± 2.5	+2.6 ± 1.2	
Insulin (pmol/L)	Baseline	26.1 ± 5.4	22.6 ± 4.6	17.6 ± 3.2	22.4 ± 1.3	0.021, 0.374
	12 weeks	26.5 ± 5.4	33.5 ± 8.4	24.7 ± 2.3	24.9 ± 2.1	
	Δ	+0.3 ± 7.5	+10.9 ± 5.8	+7.1 ± 3.7	+2.6 ± 1.7	
HbA1c (%)	Baseline	5.37 ± 0.11	5.54 ± 0.27	5.10 ± 0.09	5.11 ± 0.10	0.001, 0.823
	12 weeks	5.21 ± 0.08	5.34 ± 0.27†	5.00 ± 0.07†	4.97 ± 0.04	
	Δ	−0.16 ± 0.06	−0.20 ± 0.02	−0.10 ± 0.04	−0.14 ± 0.08	
C-peptide (pmol/L)	Baseline	374.4 ± 50.9	366.9 ± 40.5	369.9 ± 31.5	360.8 ± 29.1	0.001, 0.578
	12 weeks	470.1 ± 71.3	492.0 ± 99.2	427.6 ± 36.6	415.8 ± 31.0†	
	Δ	+95.6 ± 66.1	+125.1 ± 67.9	+57.7 ± 30.8	+55.0 ± 13.1	
sE-selectin (ng/ml)	Baseline	41.6 ± 7.1	48.9 ± 8.7	41.1 ± 2.9	39.3 ± 3.5	0.001, 0.442
	12 weeks	47.4 ± 7.1	51.3 ± 8.2	44.0 ± 2.9	46.2 ± 3.7†	
	Δ	+5.8 ± 3.7	+2.4 ± 1.6	+2.9 ± 2.2	+6.9 ± 2.0	
sVCAM-1 (ng/ml)	Baseline	748.3 ± 56.8	689.6 ± 43.9	603.4 ± 24.2	692.5 ± 47.8	0.019, 0.060
	12 weeks	771.8 ± 66.5	692.6 ± 45.8	610.8 ± 26.1	760.3 ± 59.0†	
	Δ	+23.5 ± 19.1	+3.0 ± 27.2	+7.4 ± 16.3	+67.8 ± 17.5	

Values are mean ± SEM. WG, walking group; WSG, walking + supplementation group; SG, supplementation group; CG, control group; TC, total cholesterol; HDL-C, high density lipopoprotein cholesterol; LDL-C, low density lipopoprotein cholesterol; sE-selectin, soluble E-selectin; sVCAM-1, soluble vascular adhesion molecule-1.

† Significantly different from the baseline value in the same group (Paired *t*-test, *P* < 0.05).

‡ Significantly different from the CG (One-factor ANOVA with Bonferroni adjustment, *P* = 0.007).

Δ, 12 weeks – baseline difference; w, walking; ws, walking + supplementation; s, supplementation; c, control.

## Discussion

The main purpose of this study was to examine the effects of 12 weeks of a low-volume walking exercise programme (< 150 minutes/week) combined with vitamin E supplementation on plasma TBARS and oxidised LDL concentrations in older adults. We found that plasma oxidised LDL concentrations did not decrease over the study period, but lower TBARS concentrations were observed in all intervention groups (WG, WSG, SG) relative to the control group at the end of the study period. These results suggest that 90 minutes of walking per week and/or increased vitamin E intake may protect against oxidative damage in older adults.

Oxidative stress is associated with the development of chronic disease processes such as atherosclerosis and cardiovascular disease [21,22]. The oxidative state is dependent on the equilibrium between the formation of free radicals and the action of antioxidant systems [23,24]. However, free radicals increase with advancing age [25]. Our results demonstrate that a low-volume walking exercise programme decreased plasma concentrations of TBARS (a marker of oxidative damage). This has important for public health implications, and determining the minimum activity level that is beneficial could provide practical guidance to the general public and facilitate compliance with exercise recommendations. A number of studies have reported that the majority of individuals in many countries engage in insufficient amounts of physical activity and fail to meet current physical activity recommendations [26]. Although the clinical relevance of our findings is not yet known, elevated concentrations of oxidative stress markers increase with aging and are associated with an increased risk of conditions such as cancer, cardiovascular disease and metabolic syndrome [27,28]. A recent prospective study demonstrated that insufficient physical activity was related to higher mortality rates and shorter life expectancies in both men and women [29]. Thus, a small change in plasma TBARS concentrations brought about by walking could have important health benefits.

We found that plasma TBARS concentrations were lower in the SG, WG and WSG compared with the CG at the end of the study period. Vitamin E is believed to be a beneficial for reducing oxidative stress and much attention has been paid to whether vitamin E improves the oxidative status in older adults [13]. Moreover, Vitamin E, exhibits antioxidant and anti-inflammatory effects and is able to modulate immune function [30]. The Recommended Daily Allowance for vitamin E is 15 mg (35 μmol/L) /day, which is required to maintain normal plasma α-tocopherol concentrations [31]. In Japan, adequate intake is 7.0 and 6.5 mg/day of vitamin E for men and women, respectively, to maintain the normal plasma α-tocopherol concentration of 12 μmol/L (Dietary Reference Intakes for Japanese, 2010). In our study, participants maintained their normal diet during the intervention period, and the SG and WSG received additional supplementation of 450 IU (300 mg) /day of vitamin E. The Tolerable Upper Intake Level for adults is set at 1,000 mg (2,325 μmol) /day of vitamin E; this limit is based on the adverse effect of increased tendency to haemorrhage when higher doses are taken [28]. However, the present study did not provide placebo tablets for the CG and WG. Therefore, compared with the results of other placebo-controlled studies, our results should be interpreted with caution [13,32]. It has been previously reported that both regular physical activity and vitamin E supplementation are useful for reducing lipid hydroperoxide concentrations and resting blood pressure in older adults [13]. In this previous study, the exercise intervention consisted of exercising at 50–75% of the predicted maximum heart rate for a minimum of 30 min twice weekly combined with 400 IU (267 mg) vitamin E supplementation. Our findings emphasise the potential health benefits of low-volume walking programme (90 minutes/week) and encouraging vitamin E supplementation (450 IU (300 mg) /day) for older adults. Low-volume physical activity may act similarly to, or synergistically with, vitamin E to reduce markers of oxidative damage in this population.

We found no significant changes in plasma oxidised LDL concentrations in the intervention groups over the study period. These findings are inconsistent with those of a previous study [33], which reported that long term (3 years) supplementation with 400 IU (267 mg) of vitamin E daily in healthy individuals significantly reduced circulating oxidised LDL. Whereas many studies have reported that high dose of vitamin E are poorly effective at decreasing levels of lipid peroxidation in humans [34,35]. Therefore, the shorter duration of our study might explain this difference in results [36]. Another study previously reported that high intensity training (66% of heart rate reserve, 60 minute/session, 3 times/week for 10 weeks) significantly reduced plasma oxidised LDL concentrations in older adults [6]. However, we chose to utilise a low-volume walking exercise programme because it is difficult to achieve compliance with high intensity physical activity recommendations, particularly in older adults. It would be of interest to examine a more long term exercise programme and vitamin E supplementation in a similar population to see whether this influences oxidised LDL concentrations.

Previous studies have examined the combined effects of healthy life style choices on mortality risk to assess the additive effects of such behaviours [37,38]. It is important to address this issue because combining physical activity with other healthy lifestyle behaviours (such as a healthy diet, not smoking, and only moderate alcohol consumption) appears to provide greater protection from disease than physical activity alone. In the present study, our results showed that cardiovascular disease risk markers (i.e. plasma glucose, sE-selectin, sVCAM-1, C-peptide concentrations) were significantly increased in the CG after 12 weeks compared with the baseline values; but no significant changes occurred in the WG, SG and WSG. On the other hand, the present study found that a low-volume walking exercise programme increases serum HDL-C concentrations. These observations are consistent with data from the meta-analyses [39], indicating physically active individuals have significantly higher concentrations of HDL-C compared with sedentary counterparts. Also, a meta-analysis of previous studies has suggested that higher training volume (i.e. duration × intensity) was necessary to increase HDL-C [40]. Since increased circulating concentrations of oxidised LDL and other cardiovascular disease risk markers observed in the CG these results may suggest that a sedentary lifestyle lead to accelerated atherosclerotic lesion formation. However, this is only speculation. Thus, it would be interesting to assess whether increased or decreased these parameters during a long-term period influences by their sedentary lifestyle.

The present study had some limitations. First, this study was a non-randomised trial and thus we have recruited the participants of each group at the same time. Thus, we could not strictly match the baseline value among groups. Consequently, there were significant differences in SBP among the four groups. Furthermore, the non-randomisation of participants to groups may negatively impact the significance of the present study due to the sample selection bias. Second, we are not able to speculate increased concentrations of glucose, sE-selectin, VCAM and C-peptide in the CG. In addition, intra-group analyses revealed that body mass in the S and Ex groups and BMI in the S group significantly increased after the intervention. It is possible to speculate that this may due in part to the dietary control as we did not fully control the participants’ diet throughout the study.

## Conclusion

In conclusion, this study demonstrated that 12 weeks of a low-volume walking programme (< 150 min/week) combined with daily vitamin E supplementation did not reduce plasma oxidised LDL concentrations and cardiovascular disease risk markers, but did reduce plasma TBARS concentrations in older adults.

## Competing interests

All authors declare that they have no competing interests.

## Authors’ contributions

JHP supervised data collection, performed data analysis, wrote the first draft of the manuscript, and supervised the walking programme. MM conceived the study, obtained funding, recruited participants, assisted JHP with data collection and blood analysis, edited the manuscript, and supervised the walking programme. MT was involved in the recruitment of participants, assisted JHP with data collection, and supervised the walking programme. KN, SRB, and SHK were involved in the recruitment of participants, assisted JHP with data collection, and supervised the walking programme. KS performed venous blood collection and provided guidance and assistance to JHP during the study. YN conceived the study and provided guidance and assistance to JHP during the study. All authors read and approved the final manuscript.
